# User Responses to a Humanoid Robot Observed in Real Life, Virtual Reality, 3D and 2D

**DOI:** 10.3389/fpsyg.2021.633178

**Published:** 2021-04-15

**Authors:** Martina Mara, Jan-Philipp Stein, Marc Erich Latoschik, Birgit Lugrin, Constanze Schreiner, Rafael Hostettler, Markus Appel

**Affiliations:** ^1^Robopsychology Lab, Johannes Kepler University Linz, Linz, Austria; ^2^Psychology of Communication and New Media, University of Würzburg, Würzburg, Germany; ^3^Human-Computer Interaction, University of Würzburg, Würzburg, Germany; ^4^Communication Psychology and Media Education, University of Koblenz-Landau, Landau, Germany; ^5^Department of Robotics and Embedded Systems, Technical University of Munich, Munich, Germany; ^6^Devanthro—the Roboy Company, Munich, Germany

**Keywords:** human-robot interaction, humanoid robot, presentation mode, immediacy, virtual reality, video, user evaluation

## Abstract

Humanoid robots (i.e., robots with a human-like body) are projected to be mass marketed in the future in several fields of application. Today, however, user evaluations of humanoid robots are often based on mediated depictions rather than actual observations or interactions with a robot, which holds true not least for scientific user studies. People can be confronted with robots in various modes of presentation, among them (1) 2D videos, (2) 3D, i.e., stereoscopic videos, (3) immersive Virtual Reality (VR), or (4) live on site. A systematic investigation into how such differential modes of presentation influence user perceptions of a robot is still lacking. Thus, the current study systematically compares the effects of different presentation modes with varying immersive potential on user evaluations of a humanoid service robot. Participants (*N* = 120) observed an interaction between a humanoid service robot and an actor either on 2D or 3D video, via a virtual reality headset (VR) or live. We found support for the expected effect of the presentation mode on perceived immediacy. Effects regarding the degree of human likeness that was attributed to the robot were mixed. The presentation mode had no influence on evaluations in terms of eeriness, likability, and purchase intentions. Implications for empirical research on humanoid robots and practice are discussed.

## Introduction

Among the technological achievements of the modern era, the development and advancement of robots certainly ranks among the most impressive feats. Humanoid robots are forecasted to become more and more popular in non-industrial contexts. Potential areas of application range from hospitals and nursing homes to hotels and users’ households. At the same time, many people are skeptical about the idea of sharing human life with robots (e.g., Sciutti et al., 2018; Gnambs and Appel, 2019), especially so when it comes to machines of highly humanlike appearance (Mori, 1970; Ho and MacDorman, 2010; Mori et al., 2012). As yet, however, few people have seen or interacted with a humanoid robot in real life. Currently, people’s understanding and their feelings toward humanoid robots are still mainly based on mediated experiences, such as TV documentaries, online clips or science fiction books and movies.

The aim of the present study is to compare the responses of users observing the interaction between a person and a humanoid robot presented in real-life to mediated presentation modes of varying immersive potential (VR, 3D, 2D). This comparison can assist at projecting changes in user responses once humanoid robots can be observed in everyday life. Given that much of the experimental research on humanoid robots is based on audiovisual footage rather than actual robots, our endeavor could further help at identifying systematic bias in scientific studies.

In recent years, several studies have shown that mediated depictions of humanoid robots—non-fictional or fictional—can influence people’s acceptance of real-life robotic machinery (Bruckenberger et al., 2013; Mara et al., 2013; Mara and Appel, 2015; Appel et al., 2016; Sundar et al., 2016). A handful of studies have further examined the impact of the presentation mode *per se* on people’s perceptions of human-like robots. In a comprehensive review on this subject, Li (2015) collected a mere four studies that actually compared participants’ impressions of physically co-present humanoids to those of televised video recordings (Kidd and Breazeal, 2004; Kiesler et al., 2008; Kose-Bagci et al., 2009; Bainbridge et al., 2011). While the review also includes several research efforts juxtaposing real robots with supposedly equivalent 3D animations, it has to be noted that only few of those studies actually featured comparable conditions in which all variables except for the presentation mode were held constant (Lee et al., 2006; Kiesler et al., 2008; Fasola and Matarić, 2010, also see Hoffmann and Krämer, 2013, on the comparability problem). Summarizing available evidence, it appears that real-life robots elicit more positive responses (Li, 2015; Wang and Rau, 2019) and are also associated with beneficial effects such as better learning outcomes (Köse et al., 2015) compared to robots that are observed on a screen. However, the reviewed research differs greatly in terms of the addressed variables; whereas aspects such as participants’ enjoyment, cooperative performance, or trust are explored in detail, other well-established constructs from the field of technology acceptance remain largely unconsidered. These include the eeriness concept often featured in studies on the much-discussed uncanny valley (Mori, 1970), a phenomenon that hypothesizes robots of high human realism to appear eerie or frightening (cf. Saygin et al., 2012). So far, only Paetzel et al. (2018) have studied eeriness as a function of agent embodiment and found a video recording to appear less uncanny than if the same agent was physically present. Similarly, none of the discussed studies actually investigates participants’ behavioral intentions following different types of robot presentation, leaving another worthwhile question unanswered.

The respective body of research has grown only slowly since the publication of the 2015 review (for additional work, see Kamide et al., 2014; Hoffmann et al., 2018; Keijsers et al., 2019). A recent overview article by Deng et al. (2019) focused on the impact of different physical or virtual embodiments of socially interactive robots, whereby a virtual embodiment could manifest in different manners such as an animated virtual representation of a robot, an animated virtual (non-robotic) agent, or a video of a robot. In total, the authors reviewed 65 experiments. Only 11 experiments compared more than two types of embodiment, and four experiments compared more than three different embodiments. The article concludes that according to the sparse, extant research, a physical embodiment outperforms a virtual embodiment in terms of perceived interactivity and task performance. Only six of the reviewed publications compare a live robot to a video (stream) of the same robot. Particularly in these studies, results are inconclusive. In experiments by Zlotowski (2010) and Krogsager et al. (2014), the virtual embodiment of the robot outperformed the physical embodiment. In most cases, the video is presented on a 2D screen and not compared to alternative presentation modes. Only Bainbridge et al. (2011) have compared a physically present robot either to a frontal live video of the same robot or to another condition in which this frontal video was supplemented by a second video feed recorded from above and presented on an additional screen (to compensate for the missing 3D information in the 2D video).

Since the results of many of the comparative studies mentioned above were inconclusive, we cannot yet rule out what might be called the null hypothesis, namely that there is no difference in user responses to meditated and non-meditated presentations of a robot and that, for example, the use of video substitutes as research stimuli thus allows to draw conclusions about real robots too. Even more so, we believe that most extant studies are still limited by a crucial shortcoming: By treating the robot’s presentation mode as a dichotomous variable—which can only manifest as either non-mediation (real robot) or one specific type of mediation such as 2D video—previous research offers only little insight into the theoretically relevant dimension underpinning the found effects. While several authors (e.g., Kidd and Breazeal, 2004; Bainbridge et al., 2011; Li, 2015; Paetzel et al., 2018) have suggested that the different experience of real and televised robots might be a function of observers’ perceived psychological immediacy, or social presence—i.e., considering the machine as more or less available, capable of acting and experiencing, and physically close (Lee, 2004)—this interpretation remains rather speculative.

In order to reach a more conclusive answer, we believe that a finer-grained comparison of different presentation modes is much-needed. To meet new technical possibilities that will open up for research and practice in the coming years, such a finer-grained approach must also take into account more advanced (yet increasingly widespread) presentation technologies such as 3D videos or virtual reality, which are characterized by a higher immersion potential than conventional screen-based media (e.g., Slater and Wilbur, 1997; Grigorovici, 2003; Skarbez et al., 2017) but were largely neglected in earlier studies. Following these considerations, the present study incorporates not two, but four different ways in which study participants could observe an interaction between a person and a humanoid robot: Actual physical co-presence in the live scenery, the presentation of the identical scene via a virtual reality (VR) headset, the presentation of the scene as stereoscopic video using 3D glasses, and the presentation of the scene as conventional 2D video.

Extending prior research, we integrated several constructs from the field of technology acceptance research (e.g., perceived eeriness, purchase intention) as well as the perceived immediacy of the observed human-robot interaction to our set of dependent variables. The concept of immediacy—i.e., the experienced directness of a situation and the seeming lack of mediation (cf. Davis, 2012)—has a considerable history in various fields such as theater and performance art (Auslander, 1999), digital learning, or virtual reality, where it was described as an important feature that distinguishes VR environments from all preceding technology (Psotka, 1995). Following Auslander (1999), immediacy can be experienced both in televised as well as in live real-world settings. This constitutes a conceptual difference to constructs like narrative transportation (Green et al., 2004) or presence (Heeter, 1992; Lombard and Ditton, 1997; Ijsselsteijn et al., 2001; Wirth et al., 2007; Hartmann et al., 2015; Skarbez et al., 2017), which refer to a feeling of getting involved, of “being there,” in a mediated, virtual world that is not the actual physical world. Commonly used measures for these constructs (e.g., the Spatial Presence Experience Scale, SPES, Hartmann et al., 2015) are therefore not necessarily suitable for experiments that take place completely or partially in the real physical environment.

A related concept that has been very popular in the discussion of mediated and virtual experiences is immersion. While some authors describe immersion as a psychological state comparable to transportation or presence (e.g., Witmer and Singer, 1998), many others define it—much differently—as the objective capability of a technical system to deliver compelling illusions of reality to the senses of a recipient (cf. Slater, 1999, 2003). According to the IESV aspects of immersion described by Slater and Wilbur (1997), technical systems can vary in how (1) inclusive (ref. the extent to which they shut out reality), (2) extensive (ref. the sensory modalities accommodated), (3) surrounding (ref. the width of the visual field), and (4) vivid (ref. resolution, fidelity, and color richness) they are. Consequently, immersion is usually not treated as a dependent but an independent variable whose intensity can be experimentally manipulated in order to explore its impact on other factors such as emotional responses or behavioral intentions (Waltemate et al., 2018; cf. Heidrich et al., 2019), notably often in the context of research on virtual humans as self-avatars or others’ avatars (e.g., Ijsselsteijn et al., 2006; Yee and Bailenson, 2007; Latoschik et al., 2017).

In the present study we follow this approach by comparing a non-mediated baseline condition to three differently immersive meditated robot presentation modes, whereby the immersive potential is considered low in the 2D video condition, medium in the 3D video condition and high in the VR condition. This classification is in line with previous publications in which virtual environments were described as more immersive than 3D video content and 3D in turn as more immersive than conventional video content (cf. Grigorovici, 2003). Accordingly, we assume that the participants in our experiment will ascribe the lowest degree of immediacy to the robot presented in 2D and the highest degree of immediacy to the actually co-present robot, followed by the robot presented in VR. Based on empirical evidence suggesting that physical robot embodiments outperform virtual ones (Deng et al., 2019), we further assume our other dependent variables (eeriness, likability, purchase intention) to result in more accentuated effects in the live condition than in the meditated conditions.

## Materials and Methods

### Participants and Procedure

The experiment took place in an enclosed space at the Austrian Ars Electronica Center, a museum on media art and future technologies. Our study participants were either invited in advance through social media or recruited directly on site at the museum. We *a priori* aspired a sample size of 120 (30 participants per cell) which allowed the identification of an effect size of *f* = 0.31 for an omnibus ANOVA given α = 0.05, power = 0.80. We obtained data from 120 German-speaking participants. The dependent variable data of one participant showed multiple missing values and was therefore excluded from analyses. Thus, our final sample consisted of 119 participants (52.10% women; *M*_age_ = 30.88, *SD*_age_ = 12.65, age range 17–75). For minors, consent from their legal guardians to participate in the study was obtained on site. Each of the participants was compensated with € 10 for the time spent.

Our participants’ level of completed education ranged from lower secondary school or apprenticeship (21%) to higher secondary school (34.5%) and tertiary education (38.7%), with 37.8% of respondents identifying themselves as students at the time of the experiment. When asked if they had expertise in robotics or programming, nearly half of the participants (46.2%) said they had no expertise at all, while 8% reported expertise at the highest level (indicated on a 7-point scale ranging from 1 = *not at all* to 7 = *very much*, *M* = 2.93, *SD* = 2.23). None of our respondents had ever encountered Roboy, the robot that was used in this study, prior to taking part.

At the start of the experiment, each participant was given some basic information about the robot they were about to see. After being randomly assigned to one of the four experimental conditions (live, VR, 3D, or 2D), participants observed a human-robot interaction (HRI) scene with a duration of 4:25 min. According to the condition assigned, this sequence was either mediated (via screen or VR headset) or played live in the examination room. After the sequence was finished, participants were guided to another place and completed a questionnaire that contained the dependent measures.

### The Robot

**FIGURE 1:**
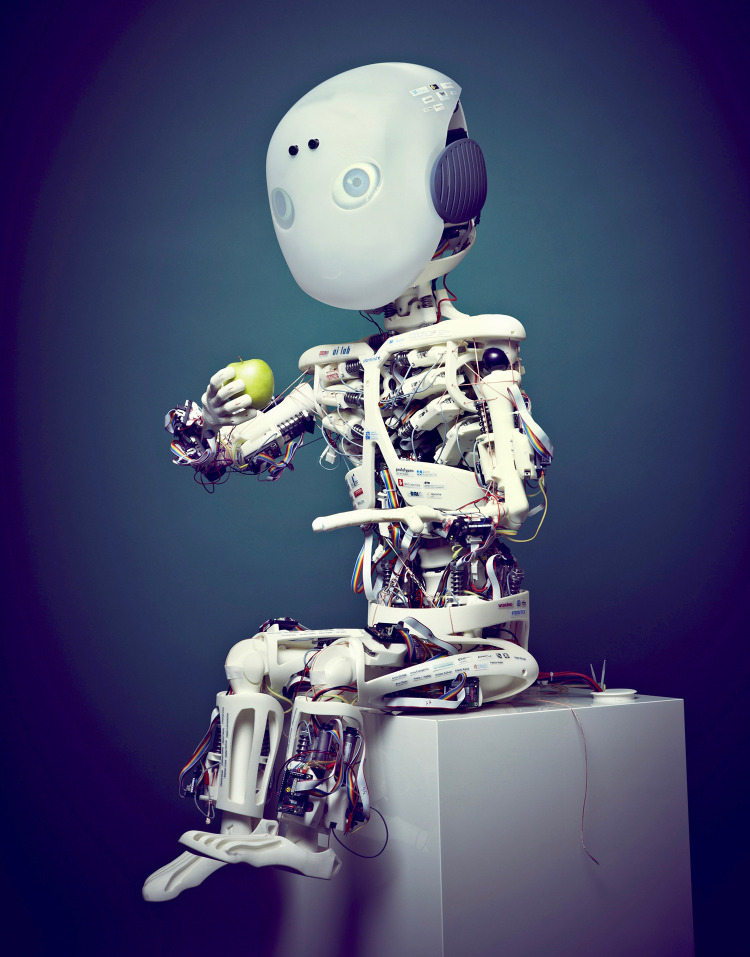
The musculoskeletal humanoid robot Roboy.

We used the humanoid robot Roboy (see Figure 1), which was created at the University of Zurich in 2013 and is now being further developed by Devanthro in collaboration with the Technical University of Munich. With a height of 1.2 meters, Roboy was designed to have the appearance of a child. Its musculoskeletal structure mimics the mechanical properties of the human body and is clearly visible to the audience. In contrast to more conventional robots, which have motors implemented in their joints, Roboy is tendon-driven, allowing for more fluent, humanlike movements. Roboy’s cartoon-like, animated face is displayed on a polymer shell using back-projection. Voice input and output is provided by two-way onboard stereo speakers built into the ears. Because Roboy was programmed to express a large spectrum of human emotions through its mimics and non-verbal behavior, the robot is considered as well applicable for the study of human-robot interaction (visit http://roboy.org for more information).

To enable a constant presentation of our experimental HRI sequence, especially for the recurring live presentations in the non-mediated condition, our study followed a wizard-of-oz approach (Kelley, 1984). This means that during the experiment the robot was tele-operated by a technician (hidden from the view of our participants) to exactly follow the scripted HRI rather than dynamically respond to real-time input. The core software components with which Roboy was controlled in the experiment are based on the open-source framework ROS (Robot Operating System).

### The Human-Robot Interaction

Participants in all conditions were confronted with a 4:25 min long human-robot interaction scene. In this scene, actor Max, who was introduced as a member of the technical staff of the Ars Electronica Center, and robot Roboy sat next to each other on stools and held a conversation in front of a neutral background. The robot used spoken language in the scene and displayed simple non-verbal behavior through facial expressions and arm gestures. The conversation followed the same script across all conditions. Its focus was on introducing possible applications that Roboy could offer to Max as a personal assistance robot, e.g., to organize and remember appointments, carry out web searches or find a birthday present for Max’s mum. You can read an excerpt from the human-robot dialog in the following (note that the scene was originally played in German):

(…)

Max: Have you already synchronized with my calendar today?

Roboy: Of course, I have already done that. (short break) Tomorrow at noon you have a meeting with Roland Aigner. (short break) Yesterday you told me that you have to buy a new servo motor before this appointment. Have you bought this servo motor yet?

Max: No, I still have to get it. We need a servo motor called “Simotics S-1FK7.” Is there an online shop that can send it in time?

Roboy: Sorry, I did not understand that.

Max: Please do a web search for “Simotics S-1FK7.” I need price and shipping information.

Roboy: “Simotics S-1FK7.” Web search in progress. (pause)

(…)

### The Four Presentation Modes

We defined four different presentation modes of varying immersive potential in which participants could experience the HRI scene described above, resulting in the following conditions:

(1). A 2D video of the HRI scene. According to the IESV aspects of immersion by Slater and Wilbur (1997), this is characterized by a low inclusiveness, a medium extensiveness, and a low surrounding. We hypothesize the lowest perceived immediacy.
(2). A 3D stereoscopic video of the HRI scene, characterized by a slightly increased inclusiveness, a medium extensiveness, and a slightly increased surrounding. We hypothesize a slightly increased perceived immediacy.
(3). A 3D stereoscopic video of the HRI scene presented via VR headset. This is characterized by a high inclusiveness, an increased extensiveness, and a high surrounding. We hypothesize the highest perceived immediacy of the mediated conditions.
(4). Live on-site observation of the HRI scene as a baseline. We hypothesize the highest overall perceived immediacy.

Details on the production and presentation of the stimuli in the four conditions include: One group of participants got to observe the HRI scene live on site. To ensure that the scene kept constant over time, a professional actor was hired who intensively rehearsed the script with the robot at first. Once the actor and the Roboy’s tele-operator were able to perform the scene identically over and over again, one live play was recorded with a stereoscopic camera pair. To keep the (perceived) distance between observer and robot constant across conditions, the two cameras were set up in the same place from which a participant in the live setting would have observed the scene, however, with an offset distance to each other in order to mimic the different viewing angles of the left and the right human eye (stereoscopic parallax).

**FIGURE 2:**
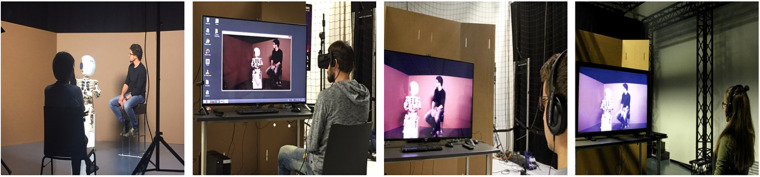
Participants observed a human-robot interaction (HRI) either live (left), or they watched the same HRI via a VR headset (second left), or on a 3D screen (second right), or on a conventional 2D screen (right).

For the 2D condition, the video feed of only one of the two cameras was used and then presented to the study participants via a 55-inch wide monitor. For the 3D condition, the video feeds of both cameras were digitally composed into a checkerboard stereo format, the standard import format for the 55-inch wide 3D monitor that was utilized for the experiment. To perceive the stereoscopic effect, participants in this condition wore LCD shutter glasses. For the VR condition, a virtual plane was created in the real-time computer graphics to cover 160 degrees of the participant’s horizontal visual field. The stereoscopic 3D video was then mapped on this plane and the scene was presented via a VR headset (Oculus Rift). Through real-time head tracking, participants could look to the left and to the right in the virtual scene. Note that participants who were assigned to the VR condition first got to watch a neutral virtual scenery to become familiar with the technology before being confronted with the experimental stimulus. See Figure 2 for visual representations of all four conditions.

### Dependent Variables

Self-reported ratings of the participants’ experience during the observed HRI served as dependent variables. Zero-order correlations between the dependent variables are shown in Table 1.

**TABLE 1:** Zero-order correlations between the main measures.

Measure	1	2	3	4	5	6
1. Perceived immediacy						
2. Human likeness	0.28**					
3. Eeriness	–0.11	−0.23*				
4. Likability	0.27**	0.35***	−0.60***			
5. Purchase intentions	0.31**	0.23*	−0.44***	0.50***		
6. Age	0.17	0.12	−0.21*	0.04	0.16	
7. Gender	0.08	0.07	0.06	0.15	–0.12	0.02

*p < 0.05; **p < 0.01; ***p < 0.001. Gender was coded 0 for men and 1 for women.

*Perceived immediacy* was derived from Davis (2012) and measured with the help of four items on a seven-point scale (“During the experiment I had the feeling that … Roboy was within my grasp,” “… I was part of the observed interaction,” “… I was right in the middle of the scene,” “… that Roboy was sitting vividly in front of me,”^1^ from 1 = *not at all to* 7 = *very much*). The scale yielded good reliability, as indicated by Cronbach’s α = 0.76.

The *human likeness* of the robot was assessed with five items on a seven-point semantic differential scale (e.g., 1 = *synthetic*, 7 = *real*; 1 = *mechanical*, 7 = *organic*, adapted from Ho and MacDorman, 2010, Cronbach’s α = 0.68).

The *eeriness* of the robot was measured with three items on a seven-point semantic differential scale (e.g., 1 = *scary*, 7 = *comforting*, as example of an inverse coded item, adapted from Ho and MacDorman, 2010), which yielded good reliability, Cronbach’s α = 0.83.

We further examined the *likability* of Roboy with five items (e.g., “Roboy is a great invention” or “I would harm Roboy if I got an opportunity,” inverse-coded, from 1 = *not at all* to 7 = *very much*), yielding acceptable reliability, indicated by Cronbach’s α = 0.68.

Finally, we were interested in participants’ *intention to purchase* a robot like Roboy. This was assessed with the help of five items (e.g., “I can well imagine using such a technology myself in the future” or “Personally, I would never spend money to buy a Roboy,” inverse-coded, from 1 = *not at all* to 7 = *very much*). Reliability was good (Cronbach’s α = 0.86)^2^.

## Results

### Omnibus Effects and Effects of Age and Gender

A multivariate analysis of covariance (MANCOVA) was conducted to identify the effects of the different presentation modes on users’ responses. Gender and age were used as covariates to partial out their effect on the results. Perceived immediacy, eeriness, human likeness, likability, and purchase intentions served as dependent variables. The MANCOVA showed a main effect of age, [*F*_(5, 109)_ = 2.44, *p* = 0.039, η*_p_*^2^ = 0.10], gender, [*F*_(5, 109)_ = 3.09, *p* = 0.012, η*_p_*^2^ = 0.12], and experimental treatment, [*F*_(15, 301.3)_ = 2.31, *p* = 0.004, η*_p_*^2^ = 0.10].

Age had a significant influence on the evaluation of perceived eeriness, [*F*_(1,113)_ = 5.18, *p* = 0.025]. Older participants described the robot as less eerie than young participants did (*r* = −0.21, *p* = 0.024). All other responses were unrelated to age. Despite the significant overall effect, gender had no influence on any of the dependent variables, all *F*s < 2.45, all *p*s > 0.098.

### Univariate Effects of the Experimental Factor

**FIGURE 3:**
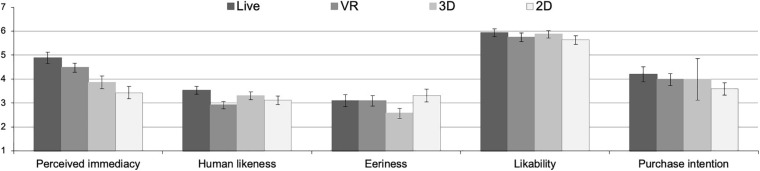
Perceived immediacy, human likeness, eeriness, likability and purchase intentions under the four experimental conditions.

Follow-up univariate analyses with perceived immediacy as the dependent measure revealed some significant differences between the presentation modes (conditions: 2D, 3D, VR, live), [*F*_(3, 113)_ = 7.10, *p* < 0.001, η*_p_*^2^ = 0.16]. Participants who saw a live HRI ascribed more immediacy to the robot than participants who observed the HRI in 3D (*p* = 0.002) or 2D (*p* < 0.001). There was no significant difference between the group who watched the HRI live and those participants who watched the HRI through a VR headset (*p* = 0.168). Likewise, no significant difference was found between the 2D and the 3D video presentation of the robot (*p* = 0.271), *M*_live_ = 4.88, *SD*_live_ = 1.32; *M*_VR_ = 4.48, *SD*_VR_ = 1.04; *M*_3D_ = 3.86, *SD*_3D_ = 1.47; *M*_2D_ = 3.43, *SD*_2D_ = 1.45. For a graphical inspection of our results, we refer readers to Figure 3.

Further univariate analyses revealed a significant overall effect of the presentation mode (2D, 3D, VR, live) on perceived human likeness of the robot, [*F*_(3, 113)_ = 2.75, *p* = 0.046, η*_p_*^2^ = 0.07]. *Post hoc* analyses indicated a significant difference between the live HRI condition and the VR condition (*p* = 0.006; *M*_live_ = 3.53, *SD*_live_ = 0.93; *M*_VR_ = 2.91, *SD*_VR_ = 0.89; *M*_3D_ = 3.30, *SD*_3D_ = 0.85; *M*_2D_ = 3.11, *SD*_2D_ = 1.00, see Figure 3). Differences between live HRI and watching a 3D video (*p* = 0.279) or 2D video (*p* = 0.088), on the other hand, failed to reach conventional thresholds of statistical significance, and there was also no difference between VR and 3D (*p* = 0.089) or 2D (*p* = 0.282). We did not find any significant influence of the presentation mode on perceived eeriness, [*F*_(3, 113)_ = 1.92, *p* = 0.131, η*_p_*^2^ = 0.05, likability, *F*_(3, 113)_ = 0.67, *p* = 0.572, η_p_^2^ = 0.02, or purchase intentions, *F*_(3, 113)_ = 0.67, *p* = 0.574, η_p_^2^ = 0.02]. All descriptive statistics can be found in the supplement.

## Discussion

Personal experiences with robots are still rare today. A recent Eurobarometer study (European Commission, 2017) revealed that 85% of EU citizens never have used a robot, neither at home nor at work. Things are quite different, though, when it comes to online videos, TV news, science fiction movies or computer games, where actual robots or fictional robotic characters appear all the more frequently. Many people—even if they have not encountered a robot in real life—are thus familiar with mediated representations of robotic technologies and may form their evaluations and attitudes toward robots based on these impressions. This is not least true for scientific studies in the field of technology acceptance and human-robot interaction, which regularly (need to) fall back on screen-based stimuli when investigating user responses, may it be due to limited resources (cf. Woods et al., 2006) or because of the restricted access to users for physical human-robot interaction, as in recent pandemic times (cf. Feil-Seifer et al., 2020). As such, the question whether effects found in studies relying on mediated depictions are transferable to the lived reality of actual, physical human-robot interactions (in the future), remained unclear due to conflicting empirical results or restrictions in the design of previous research.

The present study is the first to examine potential effects of four different digital and physical robot presentation modes in a comprehensive and highly controlled research setting. It is also the first endeavor that incorporates emerging technologies such as stereoscopic 3D recordings and virtual reality as well as previously unexplored dependent variables such as perceived immediacy, eeriness, or purchase intentions. In line with our expectations, our results on perceived immediacy show that a human-robot interaction scene played live in front of the participants outperformed watching the same HRI scene on a screen. In this regard, we extend former findings (e.g., Bainbridge et al., 2011) by showing this effect for different screen-based presentation modes (2D and stereoscopic 3D video). Interestingly, no significant difference in perceived immediacy was found between the live presentation and the presentation via VR headset. Looking only at the results of our study, it could therefore be argued that a stereoscopic presentation of a robot in immersive virtual reality might be able to serve as a reasonable substitute for a physically present robot in HRI studies, at least as long as their setup resembles the design and standardization of the current experiment (e.g., verbal interaction only). Considering the fact that the technical sophistication and immersive potential of VR headsets is constantly improving and that more and more products are launched on the market at increasingly affordable prices, virtual reality could represent an interesting option for future research designs, and by these means, also reduce problems with the technical volatility of robots in an early prototype stage.

This idea is further supported by the fact, that the experimental variations of the presentation mode, against our initial assumptions, had hardly any influence on evaluations of the robot. The only significant difference found was that the robot was assessed as more human-like in the live condition (but not in the 3D or 2D condition) compared to the VR presentation. In the overall context of the study, this is a surprising finding that encourages further research to gain a clearer picture. However, in regard to how eerie the robot was judged, how likable it appeared, or how keen participants were to purchase the robot themselves, the different presentation modes had no significant impact. What must be noted here is that our study *a priori* was designed to only find medium to larger statistical effects of the different presentation modes on participants’ evaluations and intentions. It cannot be ruled out that a study design with greater statistical power would have been able to find small significant differences that we were unable to detect with our sample size. Taking into account the results of the present study only, however, it would seem reasonable for the time being to maintain the null hypothesis, i.e., that different mediated and non-mediated presentation modes of humanoid robots do not lead to substantially different user responses. Especially in view of the rigor, the comprehensiveness and the highly controlled setup of our experiment, we believe that this is a valuable finding for the HRI research community, robotics companies, and digital media designers. However, we want to caution readers against generalizing the results of a single experiment too broadly.

Besides its strengths, we would also like to acknowledge some limitations of our study and, consequently, suggest potential starting points for future research. First, one may argue that the transferability of our results to real-world applications of humanoid robots might be limited since our study design was static, i.e., the study participants did not interact with the (physically present or mediated) Roboy themselves, but watched it interacting with someone else from a bystander’s perspective. This setting does not fully match the way user studies in the field of human-robot interaction are usually designed; however, it was the only possible way to guarantee a high internal validity of the experiment. Especially in view of the fact that many earlier studies did not come to conclusive findings or were based on only slightly comparable stimuli, we aimed at keeping all variables—apart from our experimental factor—constant in order to be able to causally attribute potential effects to the varied presentation mode only. At the cost of interactivity, we therefore opted for a short human-robot play, which we were able to present with the exact same duration, content and flow across all conditions and for all participants. We would, however, be interested to see if future studies with interactive robots would come to comparable results.

Second, as the aim of our research was to identify potential differences (or eventually similarities) between several prototypical presentation modes, we cannot make a contribution to the question how manipulations within a given presentation modality would affect user responses to a humanoid robot. For example, connections between a technical system’s vividness—introduced by Slater and Wilbur (1997) as one of four foundations of immersion—and user evaluations of a robot depicted by this system could be investigated by changing fine nuances of the resolution, color richness or fidelity of the presentation. Such aspects were not in the focus of our research. For one, the hardware principles of the displays that were used in the present study (2D monitor, 3D monitor, VR headset) made it impossible to change certain characteristics. More importantly, we deliberately chose to use the displays to account for the overall experience connected to a certain presentation mode as it would be made in everyday use. With this approach we sought for a high external validity. Nevertheless, future research could benefit from considering such more subtle technical manipulations or from incorporating measures regarding the vividness of a certain presentation modality as a potential moderating variable.

Third, we would like to address the composition of our sample. Most of our study participants were recruited among persons arriving as real visitors at the Ars Electronica Center, a museum on media art and future technologies located in Austria. Although the Ars Electronica Center is known for reaching a very wide audience, this group of people might have shared a higher-than-average interest in technologies such as robotics and virtual reality. We believe that the composition of our sample was nevertheless more diverse than frequently used “convenience samples” mainly consisting of students. In a similar vein, we are also convinced that individual differences did not confound our results, due to the randomized between-subjects design and the fact that age and gender were statistically controlled for. Future research is encouraged to carry out similar studies involving populations with rather low technical affinity or different sociocultural backgrounds.

Fourth, after having used only one robot—namely Roboy—for our study, it is not clear to what extent the results are transferable to other types of robots and their respective evaluations in different presentation modes. It must be assumed that in absolute numbers (which were not at the center of our research), other robots would not have been perceived as equally human-like, eerie or worth buying as Roboy. According to empirical studies on the uncanny valley phenomenon (Mori, 1970), highly realistic looking humanoid robots (e.g., such with silicon skin) would typically be regarded as more anthropomorphic, but also more frightening than less realistic looking humanoids such as Roboy. With its mixture of cartoonish, childlike characteristics and the exposed artificiality of its limbs, Roboy corresponds to design principles that usually resonate well with users and that can be considered as typical for a new generation of service and entertainment robots (cf. Bennett, 2019). In this respect, the robot we used may be a valid representative of many contemporary consumer robots. Nevertheless, future studies could examine whether there are interaction effects between different types of robots (e.g., in terms of visual appearance or behavioral components) and different presentation modes on user perceptions.

Taken together, we believe that the presented research makes a valuable contribution to the literature on technology acceptance, human-robot interaction, and robot embodiments. It is the most comprehensive study to date on the influence of different modes of mediated and non-mediated presentation of a humanoid robot on evaluations and behavioral intentions toward this robot. The fact that across several dependent variables no significant differences were found between a 2D, 3D, VR, or live presentation of the robot can be a relevant empirical ignition spark for research and practice. In light of the advancing progress of ever more immersive technology, we look forward to further empirical studies on the relationship between new forms of digital robot encounters and respective responses by users.

## Data Availability Statement

The datasets presented in this study can be found in online repositories. The names of the repository/repositories and accession number(s) can be found below: https://osf.io/3z4mp/?view_only=fcf116f89fe74a3ba1303bda49cf2051.

## Ethics Statement

Ethical review and approval was not required for the study on human participants in accordance with the local legislation and institutional requirements. The patients/participants provided their written informed consent to participate in this study.

## Author Contributions

MA and MM conceptualized the study. MM, CS, and RH performed data collection. RH was responsible for the technical design. MA, CS, and J-PS performed statistical analyses. MM, J-PS, ML, BL, CS, and MA discussed the results and wrote sections of the manuscript. All authors contributed to manuscript revision, read, and approved the submitted version.

## Conflict of Interest

The authors declare that the research was conducted in the absence of any commercial or financial relationships that could be construed as a potential conflict of interest.

## Footnotes

1 The original language of the questionnaire was German. Sample items listed in this section were translated into English.

2 In addition to the variables described, we also wanted to evaluate how much *agency* and *experience* participants attribute to the robot (based on two-item measures for agency and experience taken from Gray and Wegner, 2012). Since the reliability of the two indices formed turned out poor (Cronbach’s α between 0.2 and 0.4), we decided not to report agency and experience in this paper.
